# Supplementary material for: In-Depth Study on the Effects of Impurity Ions in Saline Wastewater Electrolysis
Source: Molecules. 2023 Jun 6;28(12):4576. doi: 10.3390/molecules28124576 (PMC10302785; doi:10.3390/molecules28124576)
Supplement: Supplementary file 1 [file molecules-28-04576-s001.zip › molecules-2330337-supplementary.pdf]

Supporting Information

In-Depth Study on the Effects of Impurity Ions in Saline Wastewater Electrolysis

Qicheng Pan [1,2], Peixuan Zhao [2], Linxia Gao [1,*], Huimin Liu [2,3,*], Hongyun Hu [2,3] and Lu Dong [2]

[1] College of Resources and Environment, Hubei University of Technology, Wuhan 430068, China
[2] State Key Laboratory of Coal Combustion, School of Energy and Power Engineering, Huazhong University of Science and Technology, Wuhan 430074, China; hongyunhu@hust.edu.cn (H.H.); ludong@hust.edu.cn (L.D.)
[3] Shenzhen Research Institute of Huazhong University of Science and Technology, Shenzhen 518063, China
* Correspondence: 20030007@hbut.edu.cn (L.G.); huiminliu@whut.edu.cn (H.L.)

Content of this file:

Total number of tables: 1

Total number of figures: 0

Total number of pages: 2

Table S1. Current efficiency data of all cases.

Group	Concentration of the ions (mol/L)						Current efficiency/ %
	Na^+	K^+	Ca^{2+}	Mg^{2+}	Cl^-	SO_4^{2-}	
Group 0 (Control group)	4	0	0	0	0	0	99.53
Group 1 (Effect of K^+)	3.5	0.5	0	0	4	0	99.39
	3	1					97.08
	2.5	1.5					94.97
	2	2					99.59
Group 2 (Effect of Ca^{2+})	3.99	0	0.005	0	4	0	99.11
	3.98		0.01				98.52
	3.96		0.02				97.00
	3.9		0.05				97.92
	3.8		0.1				99.04
Group 3 (Effect of Mg^{2+})	3.95	0	0	0.01	4	0	99.96
	3.9			0.05			99.77
	3.8			0.1			98.58
	3.7			0.15			98.21
	3.6			0.2			96.97
Group 4 (Effect of SO_4^{2-})	4	0	0	0	3.98	0.01	97.61
					3.9	0.05	96.34
					3.8	0.1	96.14
					3.6	0.2	99.56
					3	0.5	95.34
